# Chronic disease mortality associated with infectious agents: A comparative cohort study of migrants from the Former Soviet Union in Israel and Germany

**DOI:** 10.1186/1471-2458-8-110

**Published:** 2008-04-09

**Authors:** Jördis J Ott, Ari M Paltiel, Volker Winkler, Heiko Becher

**Affiliations:** 1 Department of Tropical Hygiene and Public Health, Medical Faculty of the University of Heidelberg, Im Neuenheimer Feld 324, 69120 Heidelberg, Germany; 2 Department of Social and Welfare Statistics, Israel Central Bureau of Statistics, Rehov Kanfei Nesharim 66, Jerusalem, Israel

## Abstract

**Background:**

Prevalence of infectious diseases in migrant populations has been addressed in numerous studies. However, information is sparse on their mortality due to chronic diseases that are aetiologically associated with an infectious agent. This study investigates mortality related to infectious diseases with a specific focus on cancers of possibly infectious origin in voluntary migrants from the Former Soviet Union residing in Israel and in Germany.

**Methods:**

Both groups of migrants arrived from the Former Soviet Union in their destination countries between 1990 and 2001. Population-based data on migrants in Israel were obtained from the Israel Central Bureau of Statistics. Data for migrants in Germany were obtained from a representative sample of all migrants from the Former Soviet Union in Germany. Cause of death information was available until 2003 for the Israeli cohort and until 2005 for the German cohort. Standardized mortality ratios were calculated relative to the destination country for selected causes of death for which infectious agents may be causally involved. Multivariate Poisson regression was applied to assess differences in mortality by length of residence in the host country.

**Results:**

Both in Israel and in Germany these migrants have lower overall mortality than the population in their destination countries. However, they have significantly elevated mortality from viral hepatitis and from stomach and liver cancer when compared to the destination populations. Regression analysis shows that in Israel stomach cancer mortality is significantly higher among migrants at shorter durations of residence when compared to durations of more than nine years.

**Conclusion:**

Higher mortality from cancers associated with infection and from viral hepatitis among migrants from the Former Soviet Union might result from higher prevalence of infections which were acquired in earlier years of life. The results highlight new challenges posed by diseases of infectious origin in migrants and call attention to the link between communicable and non-communicable diseases.

## Background

International migration is a growing phenomenon in our globalized world. But although many studies on prevalence of infectious diseases in migrants and refugees are available [1-4], mortality from these diseases is rarely considered. Even less attention is paid to mortality from chronic diseases which may be caused by viral or bacterial infectious agents [5].

A recently published study examined mortality from avoidable causes by ethnic origin in the Netherlands. After adjustment for age migrants experienced twice the risk (RR) of death from diseases of infectious origin such as pneumonia, HIV, liver cancer, viral hepatitis, tuberculosis, and chronic rheumatic heart disease compared to native Dutch. After adjustment for age, sex, marital status, urbanization level and area income the relative risk for migrants remained high at 1.50 (1.32–1.72) [6].

Infectious agents involved in non-communicable diseases considered in this paper are: Helicobacter pylori bacterium (H. pylori), Hepatitis virus and human papilloma viruses (HPV). H. pylori is classified by the International Agency for Research on Cancer as a type I carcinogen [7] and most stomach cancer and most peptic ulcers are caused by H. pylori, which produces an active chronic gastritis leading to chronic atropic gastritis [8,9]. H. pylori is assumed as a cause of duodenal ulcers in about 70% of cases and of gastric ulcer in about 60% of cases [9]. An even stronger relationship is observed for gastric adenocarcinoma with H. pylori being present in more than 80% of the cases [10].

An aetiological association with cervical cancer and its precursors is known for HPV. High-risk HPV DNA is consistently identified in cervical cancer patients [11]. Studies suggest that prevalence of HPV might be higher in Russia and other European parts of the Former Soviet Union (FSU), although the distribution of types of human papilloma virus was found to be similar to other regions of the world [12,13]. Infection in childhood and youth with Hepatitis B virus is associated with the development of liver cancer. High incidence of acute Hepatitis B and C is paralleled by high incidence of chronic Hepatitis B and C [14]. Initial hepatitis virus infection and H. pylori infection is likely to be acquired in earlier years of life [15,16] and thus it can be assumed that chronic diseases caused by infections in migrants reflect the country-specific risks that migrants bring with them. The effect of immigration from high and medium endemic areas on rising incidence of Hepatitis B virus infections in the destination country has been observed in Denmark [17], Sweden [18], and the Netherlands [19].

Since 1990 the predominant region of origin of migrants to Israel and Germany has been the FSU. Most migrants from the FSU who came to Israel are of Jewish ethnicity, particularly Ashkenazi Jews, whereas most of the migrants from the FSU in Germany are descendants of ethnic Germans who settled in certain regions of Russia during the 18^th ^Century. Both migrant groups were encouraged to migrate on the basis of common ancestry and migrated voluntarily. The study population in this investigation consists of migrants from the European parts of the FSU to Israel and of a representative sample of migrants from these regions of the FSU in Germany who arrived between 1990 and 2001.

Recent studies conducted among these migrant populations have shown a lower overall mortality compared to other Germans [20] and other Israelis [21]. However, these studies concentrated on cancer mortality [22], external causes of death [23,24], and on cardiovascular disease mortality [20].

There is little information on which to base a comparison of the prevalences of these infections in the FSU with that in Germany or in Israel. Not enough is known about infectious disease mortality in the FSU or about chronic conditions caused by an infection in order to allow us to determine the extent to which these migrants are affected by their country of origin rates. Hepatitis viruses, particularly Hepatitis B virus is more prevalent in many countries in Eastern Europe and Russia than in Western Europe and constitutes a major public health problem [25]. A study among children in Moscow shows a high infection rate of Hepatitis B and Hepatitis C viruses among patients with liver diseases such as acute hepatitis, chronic hepatitis or cirrhosis when compared to non-liver disease patients [26]. The incidence of Hepatitis B and Hepatitis C has grown since 1992 in Russia and was about 43.3 per 100,000 for Hepatitis B and 19.3 per 100,000 for Hepatitis C in 1999 with detection rates of HBsAg and anti-Hepatitis C virus being much higher among drug abusing youth and among those who experienced sexual contacts [14]. The burden of morbidity and premature mortality from Hepatitis B virus infection varies across countries within the FSU and is highest in the North West, Volga, Ural, and East Siberian regions [14,27]. A high burden of Hepatitis B is also found in St. Petersburg [28] and Uzbekistan [29]. Germany is a country with low Hepatitis B prevalence, and had a Hepatitis B incidence of 1.4 per 100,000 in 2006 [30]. A study estimated that about 84% of foreign born people in Germany originated from countries with high or intermediate Hepatitis B endemicity, among them migrants from the FSU [31]. Further estimates show that of all HBsAg carriers in Germany, 42% had a migration background, resulting in an increased risk for chronic infections among FSU-migrants and the foreign born in general [31].

Seroprevalence of H. pylori infection is also estimated to be high in Russia, particularly among the Siberian Russian population [16,32,33].

Migrants might be particularly vulnerable due to risk factor prevalence in their country of origin, as well as the assimilation stress in the new country which could increase risky health behaviour. This study assesses the mortality from infectious diseases and diseases caused by infectious agents among migrants from the FSU relative to the mortality of their destination countries, Germany and Israel. Given the size of the migrant populations relative to their destination countries, the public health impact for the receiving countries can be of considerable magnitude.

## Methods

The population studied comprises regular migrants from the FSU to Germany and to Israel who migrated voluntarily and arrived between 1990 and 2001.

Selection procedures for the cohort of migrants to Germany have been described elsewhere [20] and will be reviewed briefly here. We used data from a cohort of 34,393 migrants who arrived from countries of the FSU, mainly from the Russian Federation and Kazakhstan between 1.1.1990 and 31.12.2001 and who were aged 15 years or older at arrival in Germany. FSU-migrants are randomly distributed to federal states in Germany and the study population was randomly selected from a dataset which contains individual data of all 281,356 FSU-migrants who arrived to North-Rhine-Westphalia, Germany's most populous federal state. This random sample was assumed to be representative for all FSU-migrants in Germany [34]. The study is registry-based and utilization of these data has been approved by the Federal Ministry of the Interior of North-Rhine-Westphalia.

Data on migrants to Israel were obtained from the Israel Central Bureau of Statistics (ICBS). Individual data were assembled at the ICBS by matching electronic files of administrative records of immigration and vital registration. Release of the data for analysis in an aggregate person-years format was approved by the Data Confidentiality Committee of the ICBS. The Israeli cohort consists of all 589,388 migrants who arrived between 1.1.1990 and 31.12.2001 and were aged 15 years and older at arrival in Israel and originated in the Russian Federation, Belarus, Ukraine, Moldova, the Baltic States (Lithuania, Latvia, Estonia), and Kazakhstan. More than three quarters of them were registered as Jews in Israel, and the others were non-Jewish family members.

Persons were censored at their date of death or date of moving out of the host country. For the cohort of migrants to Germany, 31 December 2005 was the end of follow-up whereas for the cohort of migrants to Israel it was 31 December 2003. Dates and causes of death for deceased migrants in Israel were available from the ICBS for all deaths which occurred between 1990 and 2001 and in 2003. For the year 2002 causes of death were not yet available.

Causes of death were coded professionally through the official cause of death registration system at the Division of Health Statistics of the ICBS and through the Federal state office for data processing and statistics of North-Rhine-Westphalia, Germany. Before 1998 causes were coded according to the International Classification of Disease version 9, and thereafter according to version 10.

Person-years were calculated for each migrant cohort and were broken down by sex, five-year age groups, calendar year, length of stay, and immigration period. Sex-specific standardized mortality ratios (SMRs) were calculated for each migrant cohort separately using the country of destination as a reference. The SMR is defined as the ratio of observed cases and the expected number of deaths. To derive the expected number of deaths, age-, sex-, cause-, and calendar year-specific mortality rates of the German population were calculated based on mortality and population data obtained from the World Health Organization Mortality Database [35] and applied to the person-years accumulated by the migrants in Germany. The reference mortality rates for the Israeli cohort were obtained from a file which contains age-, sex-, cause-, and calendar year-specific mortality rates of all those Israelis who did not arrive from the FSU between 1990 and 2004. Expected numbers of death in the migrant cohort in Israel were calculated applying their person-years to the specific rates of the non-FSU migrant Israeli population.

SMRs were calculated for all causes of death, all infectious diseases, and for the following causes: viral hepatitis (ICD-9: 070/ICD-10: B15–B19), all malignant neoplasms (ICD-9: 140–208/ICD-10: C00–C97), and for those malignant neoplasms which are associated with infectious agents: malignant neoplasm of stomach (ICD-9: 151/ICD-10: C16) and liver (ICD-9: 155/ICD-10: C22), for males and females respectively, and malignant neoplasm of the cervix uteri (ICD-9: 180/ICD-10: C53) for females. Additionally SMRs were calculated for rheumatic fever and chronic rheumatic heart disease (ICD-9: 390–398/ICD-10: I00–I09).

All 95% confidence intervals were calculated by the exact method for number of death < 30 and by Wald-approximation for 30 or more observed deaths [36].

To estimate the effect of lengths of residence on mortality from viral hepatitis, stomach and liver cancer, regression analyses was performed using Poisson regression models for each migrant cohort and cause separately. Rate Ratios (RR) were calculated using the logarithm of the person-years as the offset term and were adjusted for sex, 5-year age-group, and calendar year of death. The covariable length of residence was categorized as less than 3 years, 3 to 5 years, 6 to 8 years, and 9 or more years of residence in the destination country.

All calculations were performed using SAS Version 9.1 [37].

## Results

Characteristics of both migrant cohorts from the FSU are presented in Table 1. The cohorts differed greatly in size with almost five million person-years accumulated by the Israeli cohort and about 340,000 person-years accumulated by the German cohort. Both cohorts were comparable regarding their countries of origin and immigration period.

**Table 1:** Characteristics of migrants from the Former Soviet Union in Israel and Germany

	**Migrants to Israel**	**Migrants to Germany**
Country of origin	Russian Federation, Russia, SU, Baltic States, Belarus, Ukraine, Moldova, Kazakhstan	Russian Federation, Russia, SU, Kazakhstan, Ukraine
Immigration Period	1990–2001	1990–2001
Immigration based on	Law of Return of 1950	German Basic Constitutional Law, Art. 116
Age at arrival	15+	15+
Number of cohort members	589388	34393
Person-years	4793089	344457
Deaths	48942 (1990–2001, 2003)	2580 (1990–2005)
Male deaths	22989	1334
Female deaths	25953	1246
Mortality follow-up	31.12.2003	31.12.2005

Table 2 shows results of the mortality analysis. The overall mortality of FSU-migrants in Germany and Israel for both sexes combined was significantly lower than that of Germans and Israelis. In both cohorts, female migrants had a greater advantage over the resident population than male migrants.

**Table 2:** Standardized Mortality Ratios (SMR) and 95% Confidence Intervals: Migrants aged 15+ from the Former Soviet Union relative to country of destination (Germany and Israel)

	Germany*			Israel**		
**Cases SMR (95% CI)**	**Males**	**Females**	**Total**	**Males**	**Females**	**Total**

All Causes of Death	1334 0.93 (0.88–0.98)	1246 0.85 (0.80–0.90)	2580 0.89 (0.85–0.92)	22989 1.01 (1.00–1.03)	25953 0.91 (0.90–0.93)	48942 0.96 (0.95–0.97)
Infectious and parasitic dis. (ICD-9: 001–139 ICD-10: A00-B99)	20 1.19 (0.72–1.83)	23 1.40 (0.89–2.11)	43 1.29 (0.96–1.74)	475 0.96 (0.87–1.05)	524 0.75 (0.66–0.83)	999 0.84 (0.77–0.90)
Viral Hepatitis (ICD-9: 070 ICD-10: B15–B19)	9 4.51 (2.06–8.56)	6 3.33 (1.25–7.36)	15 3.98 (2.23–6.57)	109 1.63 (1.35–1.96)	89 1.18 (0.97–1.38)	198 1.39 (1.25–1.53)
All Malignant Neoplasms (ICD-9: 140–208 ICD-10: C00–C97)	409 1.01 (0.92–1.11)	299 0.79 (0.71–0.89)	708 0.91 (0.84–0.98)	6498 1.21 (1.19–1.24)	7567 1.15 (1.13–1.17)	14065 1.18 (1.16–1.20)
Stomach (ICD-9: 151 ICD-10:C16)	37 1.44 (1.04–1.99)	31 1.40 (0.98–1.99)	68 1.42 (1.12–1.80)	585 1.80 (1.71–1.88)	491 1.83 (1.74–1.91)	1067 1.81 (1.75–1.87)
Liver (ICD-9: 155 ICD-10:C22)	18 1.55 (0.99–2.45)	12 1.80 (0.93–3.15)	30 1.64 (1.15–2.35)	188 1.35 (1.21–1.50)	181 1.29 (1.14–1.43)	369 1.32 (1.22–1.42)
Cervix uteri (ICD- 9: 180 ICD-10: C53)	-	7 0.82 (0.33–1.68)	-	-	83 1.09 (0.88–1.31)	-
Rheum. fever and chronic rheumatic heart disease (ICD-9: 390–398 ICD-10: I00–I09)	-	3 0.52 (0.17–1.62)	-	33 0.76 (0.41–1.10)	112 0.86 (0.68–1.05)	145 0.84 (0.67–1.00)

* Migrants in Germany, 1990–2005, relative to all Germans.

** Migrants in Israel, 1990–2001, 2003, relative to other Israelis.

While SMRs for all infectious and parasitic diseases were not statistically significant, they were greater than one for the migrants in Germany and less than one among migrants in Israel. However, when compared to the host populations, both sexes had an excess risk for viral hepatitis. Female and male migrants from the FSU in Germany were almost four times more likely to die of viral hepatitis than other Germans. Similarly, for FSU-migrants in Israel there was a statistically significantly elevated risk of death from viral hepatitis compared to other Israelis; however, compared to the host population the difference was not as great as in Germany with an SMR of 1.39 (95% confidence interval: 1.25–1.53) for both sexes combined, see Table 3

**Table 3:** Rate Ratios (RR) and 95% Confidence Intervals: Internal comparison among FSU-migrants in Israel and Germany aged 15+ by length of residence

	**Person-Years**		Rate Ratios* (95% Confidence Intervals)						
			Viral Hepatitis		Stomach Cancer		Liver Cancer		Rheum. fever/rheum. heart disease

Years of residence	Germany	Israel	Germany	Israel	Germany	Israel	Germany	Israel	Israel

0–2	123046	1657868	0.75 (0.14 – 3.88)	0.67 (0.41 – 1.10)	1.25 (0.63 – 2.48)	1.88 (1.53 – 2.33)	0.79 (0.29 – 2.17)	0.86 (0.63 – 1.19)	1.74 (0.97 – 3.11)
3–5	99301	1375751	1.06 (0.23 – 4.93)	1.29 (0.83 – 2.02)	0.92 (0.44 – 1.92)	1.58 (1.28 – 1.96)	0.73 (0.26 – 2.07)	0.74 (0.54 – 1.03)	1.47 (0.81 – 2.64)
6–8	64964	1024203	1.77 (0.41 – 7.53)	1.28 (0.81 – 2.01)	1.15 (0.55 – 2.39)	1.40 (1.12 – 1.75)	0.96 (0.34 – 2.67)	0.93 (0.68 – 1.28)	2.09 (1.19 – 3.67)
9+	57145	735267	1.00	1.00	1.00	1.00	1.00	1.00	1.00

For all malignant neoplasms, both sexes of migrants to Germany combined and female migrants specifically, had statistically significantly lower mortality than Germans. Both sexes of FSU-migrants in Israel show a statistically significant excess mortality risk for all malignant neoplasms compared to the population in their destination country.

Although a difference in all cancer mortality was observed between the migrant cohorts, both had statistically significant increased risks for two of the cancer sites examined which are associated with infectious agents. Among the migrants in Germany the risk of dying of stomach cancer was almost one and a half times that of male and female Germans, respectively. And among migrants in Israel the risk of dying of this cause was almost double that of other Israelis, for each sex. In both countries these differences were statistically significant. Similarly, the mortality risk of liver cancer relative to Germans was higher for both sexes with a joint SMR of 1.64 (95% CI: 1.15–2.35) relative to German rates. For the migrants in Israel the SMR was 1.32 (95% CI: 1.22–1.42) compared to other Israelis. No significant differences in cervical cancer mortality were found between migrants and the host population in Israel and Germany, respectively. For both sexes combined the mortality risk for acute rheumatic fever and chronic rheumatic heart disease for migrants in Israel was lower than for other Israelis and statistically significant. For German migrants no meaningful statement can be made due to the small number of deaths from this cause.

Table 3 shows results from the multivariate analysis which was carried out to assess mortality differences by length of residence in the host country. No clear pattern was observed for viral hepatitis and liver cancer. For cervical cancer, numbers were too small for this analysis. However, stomach cancer mortality in FSU-migrants was affected by length of residence, indicating mortality risks were reduced after lengths of residence of nine years in Israel. Migrants in their first two years of residence had almost twice the risk of dying from stomach cancer as those who lived in Israel for more than nine years. This result was not influenced by arrival cohort as no differences were found between migrants who arrived in the early or the late 1990s (result not shown). For rheumatic fever it also appears that the risk was reduced after 9 years in Israel, but here the pattern is weaker and only the category of 6–8 years in Israel was statistically significant.

* Adjusted for sex, age, immigration period

## Discussion

We found in migrants from the FSU residing in Israel and Germany a statistically significant elevated mortality risk for viral hepatitis and for some cancers which are associated with infectious agents. This contrasts with the overall mortality of these migrants, which was lower compared to all Germans and other Israelis. Similarly, the SMRs for all infectious and parasitic diseases (of which septicemia is the largest subgroup) were not significantly elevated for these migrants in Germany and even significantly lower among these migrants in Israel when compared to the host population.

After stratification by ethnicity of FSU-migrants in Israel and other Israelis, female migrants of Jewish ethnicity had a higher and statistically significant SMR for viral hepatitis compared to other female Israelis of Jewish ethnicity (result not shown). This might indicate that the non-Jewish female population in Israel (mostly Arabs) has higher viral hepatitis mortality, and including them in the reference population attenuates the SMR for female migrants from the FSU. However, for all other examined causes of death, the SMRs remain very similar when restricting the analysis to persons of Jewish ethnicity.

The lower risk of mortality from acute rheumatic fever and chronic rheumatic heart disease among FSU-migrants in Israel may reflect differential certification of causes of death in a population which has only recently arrived in the medical system so that diagnosis may not have been available. It is also possible that this is a selective effect reflecting diminished survival from this chronic disease before migration.

Although overall cancer mortality of male FSU-migrants in Germany was comparable to all Germans and lower in female migrants, a higher risk was observed for stomach cancer in both migrant populations when compared to their destination countries. H.pylori is however only one risk factor for gastric cancer and nutritional factors, such as low fruit and vegetable consumption and high intake of nitrite containing foods, also play an important role in the pathogenesis of gastric cancer [38,39].

Alcohol consumption, in particular vodka consumption which is very common in the FSU, was also found to increase the risk of gastric cancer [40]. High alcohol consumption, together with hepatitis virus infection, might also provide an explanation for the significantly elevated risk for liver cancer found in this study [41].

FSU-migrants in Germany and Israel at shorter durations of stay show elevated mortality risks for stomach cancer. An effect of length of stay was also observed in the Dutch study by Stirbu et al [6] where the mortality risk difference between recent and earlier migrants in the Netherlands was particularly pronounced in the case of conditions of infectious origin. Recent migrants of both sexes combined showed a significantly higher risk of death from the examined diseases of infectious origin combined, compared to those who resided in the Netherlands longer than 15 years.

Potential limitations of the study derive from the nature of the data. Not all chronic conditions involving an infectious agent are examined, e.g. due to the small number of cases rheumatic heart disease was not examined for FSU-migrants in Germany. The results presented here rely on the quality of official death certificate data. FSU-migrants and their reference populations were diagnosed and coded in the same official system, so a bias is unlikely here.

Since no information on prevalence and distribution of risk factors such as infections, alcohol consumption and poor diet are available, results are not adjusted for differences in these between host and migrant populations, and we cannot conclude from our data that infection alone explains the observed excess risks.

Differences might also be related to living conditions; however, there is neither information available on cause-specific mortality nor on infection rates of the Jewish or ethnic German population of the FSU which would allow specification of the mortality profile before migration and thus allow identification of selection and possible assimilation effects. In this regard, the general problem of the absence of ethnic-specific mortality data in studies using administrative data limits the potential for interpretation of the results presented here. However, further studies base on these data will aim at a comparison of mortality of these migrants with the country of origin using the Russian Federation mortality rates as a general reference.

It is unlikely that the mortality differences reflect differential treatment by the health care system. In Israel as well as in Germany, FSU-migrants are covered by universal health insurance and have access to diagnostic and medical services. Nevertheless, their health seeking behavior might be different than that of the host population, and some features of the health systems may function less adequately for migrants.

## Conclusion

This study suggests and highlights new challenges posed by diseases of infectious origin in migrants and calls attention to the link between communicable and non-communicable diseases. Infectious agents show association with a broad range of otherwise non-communicable diseases as in the case of H. pylori which can also affect organ systems outside the gastrointestinal tract such as skin, liver and heart [9].

To combat the burden of disease and mortality from these avoidable diseases, particularly elevated in minority groups, greater attention has to be paid to reduce infections, by treatment or by vaccination. Universal immunization intervention against Hepatitis B virus, particularly targeted towards children and adolescents, can limit the rapid spread of Hepatitis B in the countries of the FSU [14,28,29,42].

Public health initiatives are important to follow other risk factors most prevalent in the FSU such as alcohol and tobacco consumption. Since Hepatitis B virus is often transmitted intravenously and Hepatitis B incidence increased significantly among young adults in the Russian Federation between 1997 and 2002 [42] preventive action is also necessary regarding drug abuse and sexual behaviour.

The scale of migration has undergone major shifts in the era of globalization and likewise migration-associated classical infectious diseases have changed with changing source populations [15,43]. Liberalization of immigration policies in Western Europe may further affect international population movements with more people migrating from the FSU and other Eastern European countries. Given the intermediate to high endemicity of Hepatitis B virus in the FSU, chronic diseases driven by these agents might become of increasing public health relevance for both the receiving and sending countries.

## Competing interests

The author(s) declare that they have no competing interests.

## Authors' contributions

JJO initiated the topic, reviewed literature and wrote the manuscript. She made substantial contributions to the analysis and interpretation of data and was involved in data collection.

AMP made substantial contribution to the acquisition and analysis of data. He contributed to writing and revising the paper.

VW contributed substantially to acquisition, analysis and interpretation of data and contributed to writing.

HB supervised the study, made substantial contributions to the statistical methods used and to the analysis of data and contributed to writing.

All authors read and approved the final manuscript.

## Pre-publication history

The pre-publication history for this paper can be accessed here:


